# “They will say you want to make their home die”: A mixed methods study to assess modern family planning use in partnered South Sudanese refugee and host populations in Northern Uganda

**DOI:** 10.1371/journal.pgph.0000348

**Published:** 2022-06-30

**Authors:** Neha S. Singh, Pallavi Prabhakar, Agnes Ssali, Sylvia Namakula, Josephine Namatovu, Rogers Kapiti, Joram Kasiri, Sandra Mounier-Jack

**Affiliations:** 1 Department of Global Health and Development, Faculty of Public Health and Policy, London School of Hygiene and Tropical Medicine, London, United Kingdom; 2 Independent Evaluation and Research Cell, BRAC Uganda, Kampala, Uganda; 3 MRC/UVRI & London School of Hygiene and Tropical Medicine Uganda Research Unit, Entebbe, Uganda; 4 Independent Consultant, Kampala, Uganda; Institute of Public Health Bengaluru, INDIA

## Abstract

The unmet need for family planning among conflict-affected populations is high globally, leaving girls and women vulnerable to unintended pregnancies and poor sexual and reproductive health outcomes. Ours is the first known mixed-methods study to assess the use of modern family planning (FP) methods amongst married or partnered South Sudanese refugee and host populations in Northern Uganda and to explore differences between them. We conducted a cross-sectional survey in July 2019 which included 1,533 partnered women of reproductive age (15–49 years) from host and South Sudanese refugee communities in Kiryandongo and Arua. Qualitative data were collected in October 2019-January 2020 via 34 focus group discussions and 129 key informant interviews with refugee and host populations, health workers, community and religious leaders, health workers, local authorities and humanitarian actors. Our study did not find large differences between South Sudanese refugee and host populations in regard to modern FP use, though refugees reported somewhat poorer FP knowledge, accessibility and utilisation compared to Ugandan women. Reported barriers to FP use relate to access, quality of services, health concerns and family/community opposition, all of which emphasise the importance of men’s gendered roles in relationships, cultural and religious beliefs and lack of agency for most women to make their own decisions about reproductive health. Sexual and gender-based violence related to FP use was reported among both refugee and host populations. Additional barriers to FP use include lack of privacy at the public health facilities which reduces confidentiality, mistrust of health workers, and stockouts of FP commodities. Facilitating factors for FP use included: free government health services; the presence of well-trained health workers; and NGOs who give support to populations and conduct community outreaches. The findings of this study underscore the importance of developing and implementing tailored sexual and reproductive health information and services, especially for modern FP methods, in partnership with South Sudanese refugee and host populations in Northern Uganda.

## Introduction

The number of refugees is increasing globally, with the number reaching 26.4 million by the end of 2019 [1]. Uganda is the fifth largest refugee hosting country globally and the largest in Africa with over 1.3 million refugees largely from South Sudan, the Democratic Republic of Congo, Rwanda and Burundi [2]. Refugees are a vulnerable group worldwide because of the conflicts, insecurity, violence and poverty they often face. Globally, women and adolescents constitute the majority of refugee populations, however their essential health needs are largely not prioritised [3].

Family planning (FP) empowers people to make informed choices about the number and timing of births [4]. It is the cornerstone for achieving United Nations’ Sustainable Development Goal 3 aiming at universal health coverage, including financial risk protection and access to quality essential healthcare services [5]. Unintended pregnancies pose a health risk and carry associated healthcare costs, including the cost of antenatal and delivery services, as well as postpartum care for the mother, and routine healthcare for the infant [6]. The risk of illness and death of pregnant women and their children is related to parity, inversely related to pregnancy spacing and the timing of first pregnancies [7]. Short pregnancy intervals also predispose to childhood malnutrition. By averting unintended births and timing births properly, countries reap health and economic benefits through reduced pressure on the environment, agriculture, and other social services [8].

Despite these benefits, about 1 in 4 women (24%) in sub-Saharan Africa have an unmet need for FP [9, 10]. The Ministry of Health launched Uganda’s FP Costed Implementation Plan in 2014, with the aim of increasing the use of modern methods of FP from 26% of married women in 2011 to 50% by 2020. According to the 2016 Demographic and Health Survey (DHS) data, 67% of currently married women had a demand for FP; 27% wanted to limit births and 40% wanted to space births while only 39% reported using contraception. The total fertility rate is 5.4, meaning that the average Ugandan woman has 5.4 children in her lifetime [10]. These indices tend to be higher and worse off in rural and poorer subpopulations, and are thought to be worse in refugee populations. However, it is not possible to ascertain this populations’ FP status as they are not included in Uganda’s household surveys or health information system.

There is a plethora of studies published to date which focus on the sexual and reproductive health of refugee populations in Uganda [11–19] and Ugandan populations [20–28]. However, to our knowledge, there are no published studies focused on the sexual and reproductive health of both refugee and host populations, which is important to study given that both these populations are often living in close proximity to each other and have access to the same services, though experience different health outcomes [29]. Ours is the first known study using mixed methods aiming to assess the use of modern FP methods amongst partnered South Sudanese refugee and host populations in Northern Uganda and to explore differences between them.

## Methods

### Study setting

The study was conducted in refugee settlements and neighbouring host communities in Kiryandongo and Rhino camp in Arua in Northern Uganda. Kiryandongo refugee settlement, originally established in 1990, was re-opened in 2014 during the South Sudanese emergency and hosts a majority of refugees from South Sudan, with a small number from the Democratic Republic of Congo, Rwanda, Burundi, and Sudan. Rhino camp in Arua district originally opened in 1980, and expanded in the wake of the South Sudanese civil war to host the sudden influx of refugees into northern Uganda. Rhino camp is divided into six zones: Ocea, Siripi, Eden, Tika, Odubu, and Ofua. Rhino camp has expanded with the influx of refugees from recent conflicts to also include the Omugo Extension area, which is considered to effectively be the seventh zone, with Omugo overall now being the fourth largest refugee settlement in Uganda. According to the Uganda Comprehensive refugee response portal, by the end of 2019 there were 155,107 refugees and 891,700 host populations living in Arua and 55,594 refugees and 317,500 host populations in Kiryandongo [30].

Upon arrival in Uganda, refugees are usually documented and given land, tools for homesteading, rights to work and travel, and access to education and other basic public services with support from the Office of the Prime Minister, UNHCR, implementing and operating partners. However, despite the support provided to refugees, there are still gaps and numerous challenges with accessing healthcare, education, water, and fertile land that can support farming to improve livelihoods. A needs assessment and research scoping exercise undertaken by BRAC, an international non-governmental organisation (NGO), in Kiryandongo and Arua in February 2019 found that access to healthcare services in both the districts is diverse and uneven. Both refugee settlements contain health facilities that are officially run by the government but largely funded and staffed by NGOs, with governmental health facilities located outside the refugee settlements. Both types of facilities are public health facilities and are open to both refugee and Ugandan populations. This integrated approach for healthcare for both populations has been put in place since 2014, guided by the Uganda National Integrated Response Plan for Refugees and Host Communities and the Global Strategy for Public Health 2014–2018 of the United Nations High Commission for Refugees (UNHCR).

As Kiryandongo refugee settlement is closer to the town, refugees are much closer to the health centres and other private clinics compared to those living in Rhino camp, Arua. Refugee settlements in Rhino camp, Arua are farther away from Arua town which hinders the access of most refugees and host communities to the health services and livelihood options. This is due to high transport costs, limited ambulances and increased time taken to reach health facilities in town.

### Cross-sectional survey

#### Sampling and data collection

We conducted a cross-sectional survey in July 2019 which recruited 2,533 women of reproductive age (15–49 years) from host and South Sudanese refugee communities in Kiryandongo and Arua. The survey was designed to complement qualitative data collected as part of this study. The survey took an average of 40–45 minutes and collected information on the demographic characteristics, household expenditures, health seeking behaviour, utilisation and affordability of SRH services.

Sample size was calculated by assuming the proportions as 0.5, margin of acceptable error of 4%, design effect of 3.9 (intra cluster correlation of 0.10) and confidence interval of 95% level. The sampling was done at two stages. First, 20 villages each were randomly selected from host and refugee settlements in Arua and Kiryandongo. Second, approximately 30 women of reproductive age per village were interviewed by the team of enumerators. Respondents were recruited following a random walk in the village. The two-staged random sampling ensures that our sample is representative of the host and refugee women of reproductive age across the two study settings. Over the course of the study, the research team interviewed greater number of respondents to adjust for a potential non-response rate of 8%.

#### Data analysis

For the purpose of this study, we restricted the sample to women aged 15–49 years who have ever had sex and were married or in union, so we report results from 1,533 women (Table 1). Descriptive statistics on respondent and household characteristics, knowledge, access and utilisation of FP methods were analysed using Stata 15 software.

**Table 1:** Number of respondents by district and refugee status.

	Total	Kiryandongo	Arua
Host community	823	423	400
Refugee settlement	730	412	318
Total	1,553	835	718

### Qualitative research

#### Recruitment and data collection

We conducted focus group discussions (FGDs) and in-depth key informant interviews (KIIs) with local stakeholders (local authorities, NGOs, UN and multilateral agencies, health workers, religious leaders), as well as South Sudanese refugees living inside and outside of refugee settlements in Kiryandongo and Arua. We conducted 34 FGDs and 129 KIIs (Table 2). This number of participants was considered achievable, given practical considerations, and adequate to gain insight into the topic. This qualitative research was designed to complement the cross-sectional survey conducted as part of this study.

**Table 2:** Number of focus group discussion and key informant interviews by respondent type.

Respondents	Arua		Kiryandongo		Total	
	FGD	KII	FGD	KII	FGD	KII
Local authorities	1	3	2	3	3	6
NGOs, UN and multilateral agencies	0	8	1	4	1	12
Health workers from both refugee and government health facilities	3	9	1	9	4	18
Informal health workers (TBAs, volunteer community outreach workers)	0	4	0	3	0	7
Religious leaders–South Sudanese	0	2	0	2	0	4
Religious leaders–host population	0	2	0	2	0	4
South Sudanese refugees—male	3	13	3	13	6	26
South Sudanese refugees—female	4	14	4	14	8	28
Host population—male	3	6	3	6	6	12
Host population—female	3	6	3	6	6	12
Total					**34**	**129**

We identified South Sudanese refugees and host community members via the following two channels: (i) ongoing BRAC programmes within Arua and Kiryandongo and (ii) community representatives and local community groups. We identified local stakeholders using snowball sampling and investigator contacts. To participate, refugees needed to be of South Sudanese origin and registered within Arua and Kiryandongo refugee settlements. Local stakeholders needed to be working in or involved in governance or service provision for refugees in Arua and Kiryandongo refugee settlements. Potential participants were given an information sheet written in the vernacular language for those who were not fluent in English, fully detailing the study objectives and explaining all aspects of participation, including the right to withdraw from the research.

Qualitative research procedures included a member of the research team introducing the respondent to the study, its objectives and the KII or FGD to take place. If they agreed to take part, the study team members obtained informed consent from the respondents through a written consent form. After obtaining consent, study team members conducted KIIs or FGDs following a semi-structured interview guide in English or relevant local languages depending on interviewees’ preferences, using interpreters if needed to facilitate translation. KIIs and FGDs were audio recorded and reflective notes were recorded by the researchers. KIIs and FGDs were conducted in a private location convenient for participants, and in quiet environments away from clinical areas for health workers. KIIs lasted between 45–60 minutes, and FGDs lasted between 50–90 minutes. South Sudanese refugees and host populations were compensated with 29,000 UGX (approximately $8 USD) for their time to participate in the study.

South Sudanese refugees and host populations were asked about their experiences of sexual and reproductive healthcare access and utilisation, and local authorities and stakeholders were asked about various aspects of governance or provision of sexual and reproductive health services to refugee and host populations. Interview topic guides were developed for this study after pilot testing with respondent groups.

#### Data analysis

**Fig 1:**
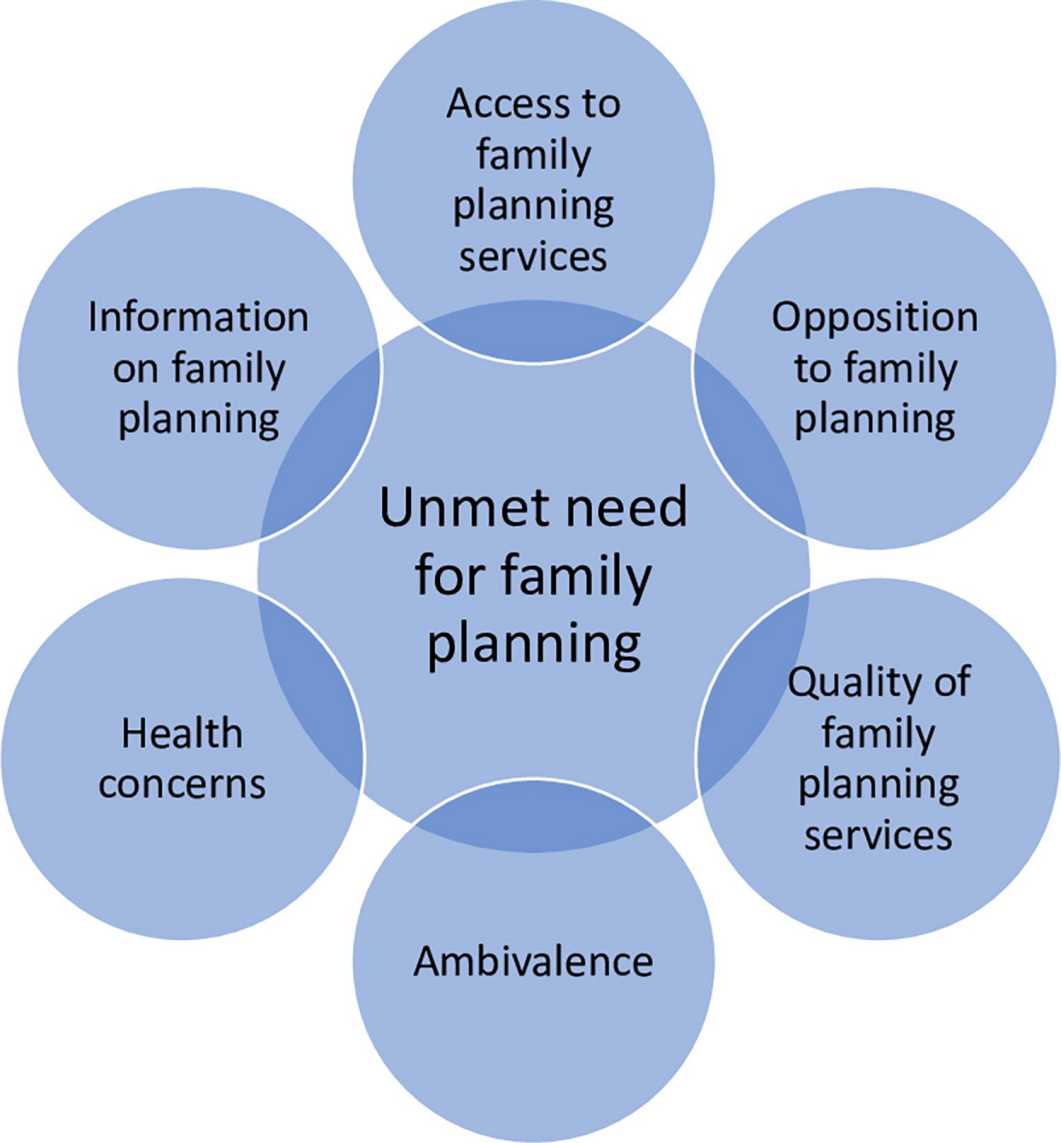
Analytical framework adapted from Mosley’s conceptualisation of research for unmet need for family planning [33, 34].

Interviews were transcribed verbatim and analysed thematically in NVivo 12 software using the following stages outlined by Braun and Clarke [31]: data familiarisation, coding and theme identification and refinement. Analytical rigour was enhanced by NSS, AS and SMJ discussing coding approaches and data interpretations. Interviews were coded using a framework approach whereby a priori and emerging themes were applied, and then data were revisited using the analytical framework (Fig 1).

We followed Noble and Smith’s recommended steps to enhance the validity and reliability of qualitative data collection and analysis, including accounting for personal biases, frequent communication with all researchers in the study team, and ongoing critical reflection of methods to ensure sufficient depth and relevance of data collection and analysis [32].

### Data triangulation and synthesis

We triangulated qualitative and quantitative findings and synthesised them according to an analytical framework (Fig 1) that we developed, which is adapted from Mosley’s conceptualisation of reasons for unmet need for FP [33, 34].These reasons relate to: (i) access to FP; (ii) information on FP; (iii) health concerns; (iv) opposition to FP; (v) quality of FP services; and (vi) ambivalence, which is defined as “unresolved or contradictory feelings about whether one wants to have a child at a particular moment” [35, 36].

### Ethics

We obtained ethical approval from the institutional review boards (IRBs) at Makerere University School of Public Health, Uganda, and the London School of Hygiene and Tropical Medicine, UK. The study was also registered with Uganda National Council of Science and Technology (UNCST) with the reference number SS296ES. Written informed consent with signatures from literate participants and a thumbprint from illiterate participants along with a witness’ signature was obtained from all study participants, and from the parent/guardian of each participant under 18 years of age.

## Results

A total of 1,533 women were interviewed in the cross-sectional survey in July-August 2019, of whom 730 were South Sudanese refugees and 823 were host nationals living in host communities. The characteristics of the respondents and their households are provided in Table 3 and compared with data from the Ugandan national 2016 DHS. Nearly twice as many refugee respondents lived in households of nine or more people compared to host communities (30.4% vs 16.8%). Education was poorer among refugees than hosts, but education rates in both populations compared unfavourably to the national average, with 19.7% of refugees having had no education, compared to 13.7% of host women and 9.6% of women nationally. However, there was a higher proportion of host households in the poorest wealth group compared to refugee households.

**Table 3:** Respondent and household characteristics from the cross-sectional survey.

	Host (n = 823)		Refugee (n = 730)		Total (n = 1533)		p value	Data from 2016 Uganda Demographic and Health Survey (DHS)
**District (%)**	n	Mean	n	Mean	n	Mean		
**Kiryandongo**	423	51.4	412	56.4	835	53.8		
**Arua**	400	48.6	318	43.6	718	46.2	<0.05	
**Age (in years)**	823	25	730	26	1533	26	ns	% age of female population
15–19 years	199	24.2	129	17.7	328	21.1		9.6
20–29 years	408	49.6	396	54.2	804	51.8		16.0
30–39 years	166	20.1	188	25.8	354	22.8		10.3
40–49 years	50	6.1	17	2.3	67	4.3	<0.001	6.3
**Respondent marital status**								% of women 15–49
Married or living as married	707	86.0	424	58.1	1131	72.8		60.6
Married but not living with husband	56	6.8	280	38.4	336	21.6		13.5
In-union and not living with boyfriend	60	7.3	26	3.6	86	5.5	<0.001	
**Highest level of education**								% of women 15–49
No education	113	13.7	144	19.7	257	16.5		9.6
Primary	555	67.4	332	45.5	887	57.1		57.4
Secondary and above	155	18.8	254	34.8	409	26.3	<0.001	32.9
**Number of children in the household**	823	3	730	3	1533	3	ns	
**Engaged in paid work**	605	73.5	266	36.4	871	56.1	<0.001	
**Household size**								% of households
1–2	72	8.8	10	1.4	82	5.3		25.1
3–4	234	28.4	116	15.9	350	22.5		28.9
5–6	223	27.0	186	25.5	409	26.3		24.5
7–8	156	19.0	196	26.8	352	22.7		13.7
9+	138	16.8	222	30.4	360	23.2	<0.001	7.8
**Wealth quintile**								
1 (poorest)	196	23.8	115	15.8	311	20.0		
2	178	21.6	141	19.3	319	20.5		
3	169	20.5	166	22.7	335	21.6		
4	134	16.3	163	22.3	297	19.1		
5 (wealthiest)	146	17.8	145	19.9	291	18.8	<0.001	
**Household head**								% of households
Female	91	11.1	463	63.4	554	35.7		31.0
Male	732	88.9	267	36.6	999	64.3	<0.001	69.0
**Household Expenditure during last month (UGX)**	823	197,023	730	207,312	1,533	201,860	<0.10	

We now apply our analytical framework to our findings, describing use of modern FP methods and using the lens of reasons for unmet need for FP (Fig 1) to explain South Sudanese refugees’ and Ugandan populations’ attitudes toward and use of FP.

### Information on FP

#### Ugandan and refugee populations’ knowledge of FP

The majority of refugee (71.1%, n = 730) and host (76.4%, n = 629) respondents reported knowledge of FP methods (Table 4). Compared to refugee women, more Ugandan women reported using any method including FP to delay or avoid pregnancy (33.7% vs 24.7%, p<0.001) (Table 4), which was also confirmed by a midwife: *‘Mostly we have the Nationals [Ugandans] who are actively involved in family planning*. *Refugees*, *ah*, *ah*. *They are there but few in number*” (KI126). The most popular forms of contraception by both groups included hormonal injections (31.7%, n = 145), hormonal implants (20.1%, n = 92), and male condoms (18.6%, n = 85).

**Table 4:** Results from cross-sectional survey on modern family planning knowledge and use amongst partnered South Sudanese refugee and Ugandan women in Kiryandongo and Arua, Northern Uganda.

	Host (n = 823)		Refugee (n = 730)		Total (n = 1,553)		p value
	n	%	n	%	n	%	
**Reports awareness of family planning**	629	76.4	519	71.1	1,148	73.9	<0.05
*Of which*							
Attended family planning training/awareness	192	30.5	82	15.8	274	23.9	<0.001
Attended training at:							
Health facility	167	87	66	80.5	233	85	ns
NGO Offices	2	1	11	13.4	13	4.7	<0.001
Hospital	12	6.3	0	0	12	4.4	<0.05
Has family planning discussion with partner	283	45	176	33.9	459	40	<0.001
Decided on number of children with partner	362	57.5	257	49.5	619	53.9	<0.001
**Reports using any method to delay or avoid pregnancy**	277	33.7	180	24.7	457	29.4	<0.001
*Of which*							
Contraceptive method used:							
Oral contraceptive pill	19	6.9	15	8.3	34	7.4	ns
Hormonal injection	101	36.6	44	24.4	145	31.7	<0.01
Intra-uterine device	2	0.7	0	0	2	0.4	ns
Hormonal implant	78	28.3	14	7.8	92	20.1	<0.001
Male condom	49	17.8	36	20	85	18.6	ns
Female condom	1	0.4	18	10	19	4.2	<0.001
NuvaRing	2	0.7	0	0	2	0.4	ns
Emergency contraceptive	2	0.7	0	0	2	0.4	ns
Withdrawal	2	0.7	0	0	2	0.4	ns
Periodic abstinence/rhythm	5	1.8	6	3.3	11	2.4	ns
Lactational amenorrhea	14	5.1	46	25.6	60	13.2	<0.001
Non-vaginal sex	0	0	1	0.6	1	0.2	ns
**Reports having to buy modern contraceptive methods**	41	14.8	5	2.8	46	10.1	<0.001
Pays for buying contraceptive methods							
Husband/Boyfriend	13	31.7	2	40	15	32.6	
Respondent	28	68.3	3	60	31	67.4	ns
**Reasons for not using contraceptives**							
Religious	14	1.7	11	1.5	25	1.6	ns
Husband/Family against	210	25.5	273	37.4	483	31.1	<0.001
Contraceptive not available/ accessible	11	1.3	38	5.2	49	3.2	<0.001
Desire for child	116	14.1	143	19.6	259	16.7	<0.001
Culturally inappropriate	39	4.7	86	11.8	125	8	<0.001
Hampers or disrupts health	136	16.5	73	10	209	13.5	<0.001
**Reports knowing that she can get a STI by not using condoms**	778	94.5	636	87.1	1,414	91.1	<0.001
**Reports knowing that she can get pregnant if not using contraceptive methods**	734	89.2	599	82.1	1,333	85.8	<0.001
**Rating the family planning facility at the health centre in the refugee settlement**							
Don’t know/Refused/Other	13	10.5	128	17.7	141	16.6	<0.05
Facility not accessible/available	1	0.8	1	0.1	2	0.2	ns
Poor	0	0	20	2.8	20	2.4	<0.10
Below average	2	1.6	117	16.1	119	14	<0.001
Average/Satisfactory	16	12.9	146	20.1	162	19.1	<0.10
Good	84	67.7	305	42.1	389	45.8	<0.001
Excellent	8	6.5	8	1.1	16	1.9	<0.001
**Rating the family planning facility at the Host Health centre**							
Don’t know/Refused/Other	138	19.8	5	26.3	143	20	ns
Facility not available	6	0.9	0	0	6	0.8	ns
Poor	8	1.1	1	5.3	9	1.3	ns
Below average	25	3.6	5	26.3	30	4.2	<0.001
Average/Satisfactory	115	16.5	2	10.5	117	16.3	ns
Good	380	54.5	5	26.3	385	53.8	<0.05
Excellent	25	3.6	1	5.3	26	3.6	ns

Women also noted the importance of making informed choices, including delaying sexual activity with their partners if they are not able to obtain FP commodities: “I just dodge him [her husband], if I did not get the medicine [FP] I just dodge him, I know the way to dodge him to avoid that pregnancy” (FGD13, Ugandan female).

Respondents also noted that FP is more acceptable to more educated women and to refugees who have spent more time in Uganda.

*Except if you find someone who is at least educated like these ladies here and they understand what is family planning. But other [less educated] community, even talking to them about family planning it is like seeing shy people*.

(FGD26, male refugee leader)

*But the refugees at least totally they have a very poor attitude about it [FP]. Mm, so, the attitude between Nationals [Ugandans] and Refugees, it is the refugees who completely have no, they do not have that zeal to get family planning. Though there are those few who have stayed like I have said those who came in the 90s, at least they have stayed for some time in the country. They can buy the idea of family planning.*

(KI121, health worker)

#### Positive role of health education and engaging local champions

Respondents widely agreed on the positive influence of health education provided at the community and facility levels on FP knowledge and use in both refugee and host communities. The majority of refugees (80.5%, n = 66) and Ugandan women (87.0%, n = 167) reported attending FP information sessions at health facilities, whereas 13.4% of refugees (n = 11) reported attending these sessions at NGO offices, and 6.3% (n = 12) of Ugandan women reported doing so at hospitals (Table 4). In particular, key informants agreed on the benefits of FP service providers engaging in two-way dialogues with communities to facilitate open question and answer sessions about FP (KI113, NGO).

*We are talking to the women, the pregnant women, the women in reproductive age about their health. Because we realise that they did not have a lot of information about their health. They think that you can get pregnant… and you produce twenty people, twenty children, one after the other. But when we went for discussions, and we have programmes where [we are] technically leading explanations to the women. When they know that the uterus has limited spaces on which the baby can plant, and any additional babies will now start to implant in the scars of the previous ones, the women start to think a lot. They say ‘eeh this is serious.’ So, you can manage to have maximum of five spaces, any other pregnancy will be in the scars of the previous ones. Then the women start to say this is serious. No one had ever told some; so, because the thinking has been that aah health workers knew that; maybe [they] thought that the women who are not educated, they cannot understand these things*.

(KI119, multilateral organisation)

*Now, these Peer Educators and the Village Health Teams [community health workers] are the very people helping us to transmit the knowledge in the community*.

(KI109, NGO, Arua)

More Ugandan women (45.0%, n = 183) compared to refugee women (33.9%, n = 176) reported having FP discussions with their partners (Table 4). Almost half the respondents (47.3%, n = 734) reported deciding on number of children to have with their partner, with more Ugandan women reporting this (53.6%, n = 441) compared to refugees (40.1%, n = 293) (Table 4). Community leaders, local authorities, health and NGO workers also emphatically agreed on the importance of engaging men and local champions with influence such as religious and other local leaders to increase the acceptability, knowledge and use of FP in both refugee and Ugandan populations.

It has reduced on the biasness the negativity about the [FP] services… *we have engaged the Peer Educators*, *the Village Health Teams*, *the local leaders*, *the cultural leaders and religious leaders*. *Okay*? *Then the politicians around there… the people who heads the families*.

(KI109, NGO, Arua)

Respondents also emphasised the importance of male engagement groups and working with male role models as they “know their community much better” than external actors and “give their testimonies” to influence and educate refugee and host communities on a range of gender and health issues including FP (KI117, NGO, Kiryandongo).

### Access to FP services

Most refugee women reported using health facilities in refugee settlements compared to governmental health facilities. About two thirds of refugee women (63.3%, n = 459) reported health facilities in refugee settlements as being satisfactory or better, compared to 18.9% of refugee women (n = 137) reporting them as below average or poor (Table 4). Interestingly, host women rated their satisfaction with health facilities in refugee settlements more highly compared to governmental health facilities (87.1% vs. 74.6%, p<0.001) (Table 4). However, both refugees and host populations widely agreed that main factors affecting their access to and use of FP services included the distance to the health facility, as well as the long queues from “morning to nine hours or evening” that they encountered at governmental health facility that made going to private clinics more appealing (KI2, female refugee).

*Because aah if you ask them, they say aah we prefer going to the private clinic*

*The queue is not there*.

(KI114, NGO, Arua)

*And how I got to know that [the popularity of private providers] is because… the organisation that has been running private family planning units here have been coming to me and they have many clients coming from the settlements seeking their services here*.

(KI104, local government authority)

Respondents also reported lack of privacy in health facilities “may expose” women seeking FP (KI114) who are often seen as a “prostitute” by refugee communities (KI125, health worker; FGD31, community health worker). Accordingly, many respondents reported being more comfortable seeing FP services from private clinics.

*They know they can get it [FP products] in the health facility. But because of stigma they fear going so, they prefer the private clinics because they are private… and there is a privacy, there’s confidentiality because people are few*.

(KI114, NGO)

Cost of FP was not reported as a barrier due to FP products from governmental health facilities being “free of charge” (KI109, NGO; KI4 refugee female; KI63 refugee male). However, due to aforementioned issues relating to privacy, distance, and overcrowding at governmental health facilities, as well as stock outs of some FP commodities, several respondents reported paying fees for FP through pharmacies or private providers, which are present in both refugee settlements and surrounding areas. In the survey, a minority of respondents reported paying for FP commodities, though more Ugandan women reported paying compared to refugees (14.8%, n = 41 vs. 2.8%, n = 5, p<0.001). Of the women who reported paying for FP commodities, the majority of women (67.4%, n = 31) reported that their partners paid for this cost. The average cost for FP commodities reported by host women was UGX 3,787 and for refugee woman was UGX 6,600 (p <0.05).

### Quality of FP services

#### Availability of FP methods

Stock outs of FP commodities, in particular at governmental health facilities, were reported by both FP service users and providers in Arua and Kiryandongo (KI111, NGO; KI121 health worker; KI65 male refugee; KI31 Ugandan female). Few survey respondents reported unavailability of FP commodities as a reason for not using them, though more refugee women reported this (5.2%, n = 38) compared to Ugandan women (1.3%, n = 11) (Table 4). An NGO which charges for FP commodities during mobile outreaches reported these stock outs as part of their motivation to provide FP in Arua and Kiryandongo.

*But also*, *the other factors are more of system factors beyond what we do*. *Facilities having stock outs*, *maybe there are no providers*, *government providers who have been trained to provide contraception*. *That is where we are now coming in trying to bridge that gap*.

(KI114, NGO)

In particular, several FP providers in governmental and NGO facilities reported running out of “those emergency [contraception]pills,” (KI118, NGO), which meant that populations “mostly… go to [private] clinics” to obtain them (KI113, NGO).

Interruption in FP supplies was also reported as a barrier to accessing FP services from governmental facilities and a reason for those who can afford it to get FP commodities from private providers.

*Yeah, that is now what disturbs people at times I may start, now if I start [FP] today, maybe it will be after six months or what? I don’t know, when I go [to the health facility] they say it is not there we don’t have the [family] planning so you come next week, next will come, you come still you cannot get it there, so these people say that these people are saying that let us do family planning we go to the hospital but we did not get, but a person who has interest will maybe squeeze herself and get something [privately]*.

(KI45, female refugee Christian leader)

#### Provider-client relationships

The majority of women reported that health workers in governmental health facilities were often unwilling to give them FP commodities without their husband’s permission, with a female refugee in Arua reporting that if a woman’s husband is in South Sudan or living elsewhere for work, “the [governmental] health centre will like tell them ‘you go to your leaders in the community to write for you a chit [note] so that we prove that really your husband is not there’” (KI116, female NGO community volunteer). However, a few women reported that health workers especially in private health clinics “cannot ask about your husband, if you wish [to pay for FP] they can just give you” (FGD5, refugee female).

Several respondents reported not trusting health workers and finding interactions with them “rude and harsh” (KI29, Ugandan female), with a refugee male going further to state that “sometimes in this society people do not believe in the health workers injecting the women [with FP]… they think they [health workers] want to kill our people.” (KI70).

An NGO worker partially attributed health workers’ interaction style to language differences and reported trying to address the issue through trainings with health workers.

*P: … you know at times they [health workers] may sound rude, but that is how they are*

*I1: Why do you think they sound rude? or why do people think they sound rude?*

*P: I think the environment they have been living in*

*I1: Okay*

*P: It was a harsh environment and also communication gap*

*I1: What do you mean by communication gap?*

*P: Aah at times we have differences in our languages. How we speak, I feel it is a little different because when I first came here [Northern Uganda] also, I found, eeh, they don’t know how to approach even greetings alone!*

(KI117, NGO)

### Health concerns related to FP

Despite 16.5% (n = 136) of Ugandan women and 10.0% (n = 73) of refugee women reporting not using modern FP methods because they hamper or disrupt health in the survey (Table 4), nearly all respondents interviewed reported refugee and Ugandan populations’ prevailing health concerns related to FP. These were often rooted in false myths and misconceptions, which hindered their use of FP.

*Family planning is still a big challenge, there are so many myths and misconceptions about family planning and that is deterring people from coming for family planning… yes, people think family planning is associated with so many bad things. Whenever they get these mild side effects, actually, one thing I have learnt; when a woman is on family planning, she does not want to fall…she does not want to get any sickness. There was a woman who just got a rash due to any oil [and] she will associate it to family planning. Even if the husband stresses her, she loses weight, she will associate it to family planning*.

(KI132, hospital manager)

Specifically, several Ugandan and refugee respondents in addition to health and NGO workers reported a range of side effects of FP. These included proven side effects of FP such as abnormal bleeding and pain, as well as perceived side effects such as foetal abnormalities including those whose “head is very big, [and] the body is very small” (FGD27, male refugee leader), wombs “getting rotten” and leading to cervical cancer (FGD25, female Ugandan village leader; KI130, health worker) and infertility.

*Yes, they [Ugandans and refugees] don’t believe in the effectiveness [of FP]. They look at the risk also because at times the people who have used [FP as] a woman fails to conceive*.

(FGD27, male refugee leader)

*That is why women are now suffering because when I inject [contraceptives] today, like me I normally use for three months, but when I use it, it always disturbs me I feel pain, I bleed, and I had to stop*.

(FGD26, female refugee leader)

### Opposition to family planning

#### Religious opposition

Both refugee and Ugandan communities, including religious leaders, noted the key role that Islam and Christianity plays in hindering the use of FP, though only 1.6% of survey respondents (n = 25) reported religion as a reason for not using modern FP methods.

*P: No no I will not encourage you to put on condom because it is not allowed in the religion [Islam]*

*I: It is not allowed in the religion?*

*P: It is not allowed yes you know God said he has not provided for us this sperm to spoil*

*I: Ehh it is not for wastage?*

*P: Yes, it is not for wastage it has a use*

(KI100, Muslim leader)

*Refugees are complicated. I think uhmm they are not so much into family planning. And just by virtue of their [religion], most of these Dinka are Arabic. I think uhmm Arabic Muslims, family planning is not in their teaching*.

(KI132, health worker)

The authority that religious leaders command in the community influences the choice to seek FP, as was reported in a FGD with Ugandan village leaders: “If you decide to go for family planning, the Father [Catholic priest] will say you have killed the children because God said, ‘you produce! Even 100 [children]*’*”‘ (FGD25).

Some respondents noted that while training community leaders in the importance of FP was useful, religious leaders were being missed out despite them having greater influence on communities’ decision-making:

*Because sometimes you know, church leaders speak bigger. People consider them to be spiritual leaders, so, if we can also pick them and give them the knowledge alongside other leaders in the settlement, I think it can work much better. Even in our own secondary schools*.

(FGD27, refugee leader)

#### Cultural opposition and the role of conflict on FP use

Several respondents noted that “cultural tradition does not allow them [refugees and Ugandans] to go for family planning” (KI122, health worker). FP was viewed as contrary to both Ugandan and South Sudanese culture, with more refugee survey respondents noting modern FP as being culturally inappropriate compared to Ugandan women (11.8% vs 4.7%, p<0.001) (Table 4).

“*Ehh our culture does not support family planning*. *Because the African culture said*, *you must produce the children”*

(FGD25, Ugandan village leader).

*Let me have a say on this. Like my colleague said, ahh [modern] family planning, it is not a new thing this is something people heard, people went through, it is there in South Sudan. And the resistance [in South Sudan] is still the same like what is happening here at the refugee settlement. So, like he said, culture is deeply involved in this [opposition to family planning]*.

(FGD27, refugee representative)

The majority of respondents also noted the role of the conflict in South Sudan hindering refugees’ FP use, as “they feel they should deliver the number of their people who have died in the war” (KI122, health worker). In particular, more recently resettled South Sudanese refugees were reported as being more reluctant to use FP because “they have lost very many people, so they do not want to [use it]” (KI121, health worker).

The desire amongst some South Sudanese populations to replace those lost in the war was so great, that a female refugee representative reported if a woman’s husband is away for a few years, then it is culturally acceptable for his relative to impregnate her:

*They said that most of the children have died in the war so they need to replace, that is why others even if the husband is in South Sudan and you don’t communicate with the woman if it takes more than three years, another person who is here related [a relative] to the husband will go with this woman and produce the children for that man. Until you marry your own wife, even if you have a wife, you go with this woman after producing the children belong to that man. The children are nicknamed [given] the name of that man [and] even when they go to school, they will write the name of that man*.

(KI41, female refugee representative)

Compared to Ugandan women, a higher proportion of refugee women surveyed also reported desire for a child as a reason for not using FP (19.6% vs. 14.1%, p<0.001).

#### Male opposition and perpetration of violence

Male partner objection to FP was a significant barrier to FP use highlighted by all respondent groups. A higher proportion of refugee women reporting male or family opposition to using modern FP methods (37.4%, n = 273) compared to Ugandan women (25.5%, n = 210) (Table 4).

*Participant 2: Men don’t support women in family planning. That is why you see they [women] are hiding. They want to come for [FP] injections. There was a young man who brought the woman here. The woman came without telling him. But the next day he saw, they came that they wanted to see the person who inserted that method. So, we told them it is [private provider], they took off from here, they went up to town following [private provider]. They should remove that thing. I think they removed from town. [The man said] That the lady is still young, and he is still young, they want to produce [children]. They don’t want anything like family planning. Why? He married the woman to come and produce [children]*.

*Participant 3: Most times men they now want children*.

*Participant 2: When they have 10 [children], they still want more*.

(FGD29)

*You know some men they do not want you to put on family planning they will say you want to make their home die… you will have to produce children… but when somebody sees you putting in [FP] and the person brings the information to your husband sometimes they bring problems and some men they also decide to take their [wives] back to the health centre, to where they have put it in and that nurse who put it in the wife will have problems with them*.

(KI1, refugee female)

Although men reportedly refuse their partners using FP methods, they were also reported as being reluctant to use short-term or permanent FP methods.

*Men don’t want [a] vasectomy, they know their manhood will [laughs and gives a single clap]. The fear is about losing their manhood. They say, ahh, how do you? How do you cut the wire? The problem is that…that is how they call it. They say, how do you cut the wire. Yeah! [laughs] then when you cut the wire do you expect the wire to be there*.

(KI132, health worker)

*Participant 5: Yeah you [his wife] bring condom. How can you come and talk something like sex to me! (laughs)*

*Participant 7: How can I use condom to my own wife that I have married with money?*

(FGD26, male refugee leaders)

Male desire to control female fertility and FP use often translated into the threat or perpetration of sexual- and gender-based violence (SGBV) toward their partners if their desires were not followed, with a refugee woman stating that “if you use it [FP] alone, it will bring fighting at home…and sometimes he will beat you” (KI10). A refugee traditional birth attendant further explained that South Sudanese men have fertility expectations linked to the dowry they pay when marrying: *“When you [are] a Dinka woman [and] you refuse [sex]…they [men] marry them with cows so if you refuse the man will beat you”* (KI54).

Reports of SGBV caused by FP use was also confirmed by male respondents and health workers:

*It [FP] has also led to a lot of family domestic violence*, *for example if a woman uses a [FP] method of 5–10 years without conceiving*, *now the man begins demanding for a child and ends up insulting the woman and even chasing her away hence [leading to] family break down and divorce*. (KI89, Ugandan male)

*Aaaah I think the husbands*, *the*, *men are not supportive enough to take decisions on family planning*, *[whether] she would put it [on] now*, *by the following week she will come back [saying] my husband has beaten me*, *he doesn’t need family planning*. (KI130, health worker)

### Ambivalence

Both refugee and host populations described tensions between fertility desires and challenges associated with livelihoods and being able to provide for a larger family. Despite having the right to work and being given plots of land to live and farm on when they arrived in Uganda, many refugees, especially in Arua, reported struggling with producing crops on arid land. Additionally, many host respondents also reported struggling with making ends meet, which subsequently impacted their decision-making around limiting fertility.

## Discussion

The findings of this study suggest that while Uganda has been successful in integrating refugees into the health system in Arua and Kiryandongo, access- and information-related barriers specific to modern FP methods for both refugee and host populations need to be overcome. Our study did not find large differences between South Sudanese refugee and host populations regarding modern FP use, though refugees reported somewhat poorer FP knowledge, accessibility and utilisation compared to Ugandan women. Reported barriers to FP use for both refugee and Ugandan populations relate to access, quality of services, health concerns and family/community opposition, all of which emphasise the importance of men’s gendered roles in relationships, cultural and religious beliefs and lack of agency for most women to make their own decisions about reproductive health. In particular, refugees faced greater cultural and family pressure to have children and noted the role of wanting to replace those lost in war as a reason to not use FP. SGBV related to FP use was also reported among both refugee and host populations. Additional barriers to FP use include lack of privacy at the public health facilities which reduces confidentiality, mistrust of health workers, and stockouts of FP commodities. Facilitating factors for FP use include among others: free government health services; the presence of well-trained health workers; and NGOs who give support to populations and conduct community outreach.

Our study findings are consistent with findings from a number of studies assessing FP attitudes, knowledge and use amongst crises-affected populations in both Uganda and other Sub-Saharan African countries. This includes the 2016 Uganda DHS survey which reported a similar contraceptive method mix as our study findings [10], as well as two studies reporting cultural factors, lack of knowledge, availability and accessibility impacting FP use amongst adolescent and young women in Uganda [11, 37]. Our study findings are also echoed in two systematic reviews on FP use amongst crises-affected women in Sub-Saharan Africa, which report a number of factors affecting modern FP use including accessibility and availability of FP commodities, mistrust of health workers, religious and cultural opposition, influence of husbands and communities, SGBV, and myths and misconceptions about FP commodities [38, 39]. Our findings also highlighted the increased need to get FP information and services as close as possible to the intended recipients through community-based programming, which is also similar to a study conducted with teenage mothers in another refugee settlement in Kyangwali in Uganda [40].

Our study did not find large differences between refugee and host populations in relation to utilisation of FP services, which is consistent with findings in a multi-country study that quantitatively analysed utilisation of outpatient services in refugee settlements, including in Uganda [29]. This might be explained by the Ugandan policy to integrate services for host populations and refugees, in particular in settlements near the Sudanese border where refugee settlements are no longer refugee camps (despite Rhino camp being given this name). Compared to camps, in refugee settlements refugees can move freely and access all Ugandan services, and host communities can also live there [41–44]. This Ugandan policy integrates refugees into existing villages and has the government officially take over as the direct implementing partner of all health services, some of which were already in place, and others which were newly created and made available to all. As there are no longer any refugee-specific services, therefore it is expected that access to healthcare for both refugees and hosts will be more equitable. However, our study reports unique barriers that refugee and Ugandan populations face in utilising FP services, signalling the need to design, test and implement tailored interventions to address them. More work also needs to be done to examine how quality of services change during and following integration, especially in light of our findings that women report being mistreated by health workers and refugee women face language barriers at health facilities.

SGBV has been reported among Sudanese refugees in a study conducted among men and women survivors of SGBV [45]. This study’s findings emphasised the need to integrate gendered and culturally sensitive service provision that would bring in the formal and informal health service system to support the survivors [45]. Our study indicated that SGBV usually resulted from women going against their male partners’ desire to not use FP methods and produce as many children as possible. An integration of formal and informal services from private and public facilities and interventions to address social norms by involving men, including male champions, may contribute to the reduction of SGBV among refugee communities.

Male involvement in FP has been reported as a useful avenue to increase the use of FP amongst women. A study conducted in Uganda among men indicated that most men knew about use of condoms as a FP method; however, they reported side effects such as vaginal bleeding and misconceptions about fertility in the women which affected their acceptance for their wives to use FP [46]. Our study also reported men’s concerns about side effects of FP, especially bleeding, which meant they had to incur increased costs to take care of women. This points to the need to target men and wider community members and leaders, and to share FP information in order to avert negative reactions towards their spouses and the health providers.

Our study has several limitations. The self-reported nature of data collected in the quantitative survey, interviews and FGDs is a limitation. A lack of privacy and the sensitive nature of the topic may have also created reporting bias in the survey, interviews and FGDs. Additionally, the survey did not capture FP method switching and discontinuation. Finally, given that a proportion of the refugee population arrived in Uganda in the 1990s and that there are likely to be individuals who were born to refugees in Uganda and are now of reproductive age themselves, future research should also consider birth location or first/second generation refugee status among demographic characteristics associated with modern FP utilisation.

Notwithstanding these limitations, this study is unique in being the first to our knowledge to use mixed methods data to assess the use of modern FP methods amongst married or partnered South Sudanese refugee and host populations in Northern Uganda and to explore differences between them. Given that we did not find major differences between both populations in terms of FP use, barriers and facilitators, our findings underscore the complexities of a health system dealing with a protracted crisis and fragmented funding and resources for both populations facing similar pressures on their environment and livelihoods [47]. Moreover, our study provides information for governmental, humanitarian and development actors in Uganda to better tailor their FP services for refugees and Ugandans. Our study findings also provide researchers with data to inform the development and testing of tailored strategies to improve uptake of modern FP methods in both refugee and Ugandan populations, which are research priorities outlined in a number of recent SRH research priority setting exercises [48–50].

## Conclusions

Findings from our study underscore the critical need to work with women, men, community and religious leaders as well as healthcare providers to address the multi-factorial issues contributing to the underuse of modern FP services amongst both host populations and South Sudanese refugees in Uganda. Future research and programming should work with women, their partners, and communities to develop and test co-produced interventions and approaches to improve modern FP access, availability and use that are tailored to both host and refugee populations, while ensuring that they are delivered in a respectful and culturally sensitive manner.

## Supporting information

S1 Questionnaire PLOS’ questionnaire on inclusivity in global research. (DOCX)
